# FVTF inhibits hepatocellular carcinoma stem properties via targeting DNMT1/miR-34a-5p/FoxM1 axis

**DOI:** 10.1186/s13020-025-01084-3

**Published:** 2025-03-06

**Authors:** Xiao-Cheng Cao, Jinwu Peng, Ye-Bei Qiu, Wei Zhu, Jian-Guo Cao, Hui Zou, Zheng-Zheng Yu, Di Wu, Shan-Shan Lu, Wei Huang, Hong Yi, Zhi-Qiang Xiao

**Affiliations:** 1 Department of Pathology, Xiangya Hospital, Central South University, Changsha, 410008 China; 2 Research Center of Carcinogenesis and Targeted Therapy, Xiangya Hospital, Central South University, Changsha, 410008 China; 3 The Higher Educational Key Laboratory for Cancer Proteomics and Translational Medicine of Hunan Province, Xiangya Hospital, Central South University, Changsha, 410008 China; 4 Department of Pharmaceutical Science, Medical College, Hunan Normal University, Changsha, 410081 Hunan China; 5 National Clinical Research Center of Geriatric Disorders, Xiangya Hospital, Central South University, Changsha, 410008 China

**Keywords:** Fructus Viticis Total Flavonoids, Hepatocellular carcinoma, Cancer stemness, DNMT1, MiR-34a-5p, FoxM1

## Abstract

**Background:**

Fructus Viticis Total Flavonoids (FVTF) is a novel candidate preparation that possesses anticancer activity. However, the role and mechanism of FVTF-inhibiting human hepatocellular carcinoma (HCC) cell stem properties is unclear.

**Methods:**

Liquid chromatography (LC) in conjugation with mass spectrometer (MS) was used to identify the compounds of FVTF. Tumorsphere and soft agar colony formation ability, cancer stem marker expression levels, CD133^+^ cell percentage, and a xenograft model were utilized to investigate the impact of FVTF on HCC cells stemness. PCR array and qRT-PCR were conducted to identify differentially expressed cancer stem-related genes and miRNAs between FVTF-treated and untreated HCC cells, respectively. Pyrosequencing was conducted to assess the DNA methylation level of the miR-34a-5p promoter. A luciferase reporter assay was performed to verify whether FoxM1 serves as a direct target of miR-34a-5p. Additionally, immunohistochemistry of an HCC tissue microarray was carried out to assess the expression levels of DNMT1, FoxM1, and miR-34a-5p.

**Results:**

A total of 26 compounds, including 10 flavones, in FVTF were identified. FVTF significantly reduced the ability of tumorsphere and soft agar colony formation, the levels of CD44 protein and *BMI1*, *OCT4* and *SOX2* mRNAs in HCC cells, and in vivo tumor initiation ability of HCC cells. Mechanistically, FVTF inhibited HCC cell stem properties via targeting DNMT1/miR-34a-5p/FoxM1 axis. Clinically, DNMT1 expression was inversely correlated with miR-34a-5p expression, whereas a positive correlation was noted between DNMT1 and FoxM1 expression levels, and high DNMT1 levels, low miR-34a-5p levels, and high FoxM1 levels were associated with cancer recurrence. Furthermore, a combination of DNMT1, miR-34a-5p and FoxM1 served as an independent prognostic indicator influencing both DFS and OS in patients with HCC.

**Conclusions:**

FVTF inhibits HCC cell stem properties by targeting DNMT1/miR-34a-5p/FoxM1 axis, which is associated with HCC recurrence and prognosis, and FVTF is a prospective treatment drug for human HCC.

## Background

Hepatocellular carcinoma (HCC) ranks as the fifth most prevalent cancer globally, exhibiting a 5 year survival rate of only 18%, making it the third leading cause of cancer-related mortality [1]. Notwithstanding progress in HCC therapies, the 5 year survival rate for HCC patients remains low because of recurrence and metastasis post-therapy [2]. HCC comprises a limited population of cancer stem-like cells that promote tumorigenesis, metastasis and therapeutic resistance, therefore targeting HCC stem-like cells has become an intriguing approach for curative treatment of HCC [3]. We and other research groups have confirmed the presence of cancer stem-like cells (CSLCs) in HCC [4, 5]. Consequently, it is essential to develop potent therapeutic drugs targeting HCC stem-like cells and reveal the mechanism of their anti-HCC activity.

Fructus Viticis is the dry mature fruit of *Vitex trifolia L.* and *Vitex rotundifolia L*.. It exhibits diverse pharmacological properties across multiple biological processes and is hence acknowledged as a traditional Chinese medication [6]. Fructus Viticis comprises substantial quantities of flavonoids, including casticin, isoorientin, hesperidin, luteolin, apigenin, and isovitexin. [6]. It has been reported that casticin possesses obvious anti-cancer activity in vitro and in mice [7]. FVTF (Fructus Viticis Total Flavonoids) represents a novel development from our laboratory, serving as a desirable Chinese medicine preparation derived from Fructus Viticis, and can inhibit cancer stem characteristics in various cancers (National Invention Patent number of China: ZL 201210591146.9). However, the role and mechanism of FVTF-inhibiting HCC cell stemness need to be further elucidated.

We recently screened cancer stem-related genes and miRNAs regulated by FVTF in HCC cells, and identified and confirmed that DNMT1 and FoxM1 were obviously downregulated, whereas miR-34a-5p was obviously upregulated by FVTF, indicating that DNMT1, miR-34a-5p and FoxM1 could be the targets of FVTF in the HCC cells.

DNMT1 functions as the primary enzyme that recognizes DNA methylation patterns on the parental strand and subsequently adds methyl groups to the daughter strand in newly replicated hemimethylated DNA. This mechanism is crucial for the epigenetic regulation of gene silence, since it enables the methylation of gene promoters [8]. DNMT1 is identified as a crucial element in preserving the stem characteristics of leukemia cells [9] and breast cancer cells [10]. However, whether FVTF inhibits HCC cell stemness via downregulating DNMT1 is unknown. MiR-34a-5p, a tumor suppressor, has been shown to be downregulated in multiple cancers [11]. Our prior research indicates that the low expression of miR-34a-5p increases HCC stemness [12]. However, whether FVTF suppresses HCC cell stemness by upregulating miR-34a-5p is unclear. Recent investigation indicates that miR-34a-5p increases cell apoptosis by targeting FoxM1 in pancreatic cancer cells [13]. Previous studies also reported that miR-34a-5p impedes the development of esophageal squamous cell carcinoma by downregulating FoxM1 [14]. However, whether FVTF inhibits HCC cell stemness via miR-34a-5p-regulating FoxM1 is unknown.

This research examined the function and mechanism of FVTF in suppressing stemness of HCC cells, and found that FVTF inhibits HCC cell stem properties by targeting DNMT1/miR-34a-5p/FoxM1 axis, and indicating that FVTF is a potential therapeutic agent for human HCC. We also used HCC tissue microarray to evaluate the expression and clinicopathological relevance of DNMT1, miR-34a-5p and FoxM1. Our findings indicate that alterations in DNMT1, miR-34a-5p, and FoxM1 expression is associated with tumor recurrence. Furthermore, the combination of DNMT1 and miR-34a-5p and FoxM1 serves as an independent prognostic indicator for HCC patients.

## Materials and methods

### Preparation and characterization of FVTF

The extraction and preparation of FVTF from Fructus Viticis were conducted in accordance with the patent specification (Invention Patent number of China: ZL 201210591146.9). Composition of FVTF was analyzed using an ultra-high performance liquid chromatography (UPLC) in conjunction with mass spectrometer (MS). For further information, please refer to the Supplementary Materials and Methods.

### Clinical specimens

The HCC tissue microarray (TMA) (Cat. HLivH180Su17) was acquired from Outdo BioTech (Shanghai, China), comprising 92 HCC tissues and 88 paired paracancerous tissues. The TMA facilitated the detection of DNMT1, miR-34a-5p, and FoxM1 expression. The clinicopathological characteristics of patients are detailed in Tables S1 and S2.

### Cell lines and culture

The MHCC97H and SK-Hep-1 human hepatocellular carcinoma cell lines were obtained from the Shanghai Chinese Academy of Sciences Cell Bank (Shanghai, China). Cells were cultured in DMEM (Gibco) supplemented with 10% fetal bovine serum (Gibco) in a humidified incubator containing 5% CO_2_ at 37 °C. Prior to experimental use, the verification of the cell lines’ authenticity was conducted through short tandem repeat (STR) DNA fingerprinting. Additionally, to ensure the absence of contamination, routine mycoplasma testing was performed using 4,6-diamidino-2-phenylindole staining, with consistent negative results.

### Establishment of HCC cell lines with overexpression of DNMT1 and FoxM1

MHCC97H HCC cells underwent infection with a lentiviral vector that expresses DNMT1 or transfected with pcDNA3.1-FoxM1, subsequently puromycin was employed for a selection period of two weeks. Stable overexpression of DNMT1 or FoxM1 was established in cancer cell lines, and this was validated through Western blot analysis.

### Transient transfection

50 μmol/L miR-34a-5p mimic, miR-34a-5p inhibitor and their respective negative control (Ribobio, China) were transfected into the specified HCC cells with the RiboFect^™^ CP Transfection Kit (Ribobio), respectively. 48 h post-transfection, the cells were collected and then underwent subsequent analyses.

### Tumorsphere formation assay

The in vitro stem cell-like properties of HCC cells were evaluated through the tumorsphere formation assay, following the methodology previously outlined by our group [15]. Briefly, HCC cells were inoculated at a density of 1 × 10^3^ cells per well in 6-well plates with an ultralow attachment surface (Corning Inc.) and cultured in serum-free DMEM/F12 medium (Invitrogen), supplemented with 20 ng/ml bFGF, 20 ng/ml EGF, and 20 µl/ml B27 supplement (Invitrogen). After 7 days of incubation, tumorspheres exceeding 50 µm in diameter were enumerated using a Leica DMI4000 microscope.

### Soft agar colony formation assay

A soft agar colony formation assay was conducted to assess the anchorage-independent growth potential of HCC cells, following the methodology outlined by our group [16]. In summary, HCC cells were suspended in a 0.3% agar solution (Sigma) that included DMEM medium supplemented with 10% fetal calf serum (FCS) at a concentration of 5 × 10^3^ cells/ml. A 1 ml cell suspension was subsequently layered on top of a base consisting of 1 ml of 0.5% agar in DMEM supplemented with 10% FCS, placed in 6-well tissue culture plates. Following the plating process, 1 ml of DMEM medium supplemented with 10% FCS was added to each well, with media being refreshed every 3 days to ensure optimal conditions are sustained. Following a 12-day growth period, colonies with over 50 cells were measured using a microscope.

### ***Analysis of CD133***^+^***cell population by flow cytometry***

Cancer cells were initially collected and incubated with 5% BSA (Sigma) at 4 °C for 30 min to inhibit nonspecific binding. Thereafter, PE-conjugated anti-CD133 antibody (1:100 dilution; #566596, BD Biosciences) or PE-conjugated rabbit isotype control IgG (1:50 dilution; #550617, BD Biosciences) was added to the cells and kept on ice for 30 min. Subsequent to staining, the cells were examined utilizing a FACSCanto flow cytometer (FACSCanto II, BD Biosciences). The resultant data were processed and analyzed with FlowJo 10.0 software.

### PCR array

Total RNAs were extracted from MHCC97H treated with FVTF for 48 h and untreated control cells using TRIzol reagent (Invitrogen), respectively. The differential expressional genes were analyzed by a cancer stem cell-related gene PCR array (WC-MRNA0023-H; Wcgene Biotech, China). The 2^−DDCt^ methodology was utilized for normalizing the expression levels of target genes in comparison to GAPDH. The ABI Gene Amp PCR System 9700 (ABI) was employed to conduct qRT-PCR.

### Quantitative real-time PCR

qRT-PCR was conducted to assess the expression levels of 17 cancer stem-related miRNAs, as well as DNMT1, FoxM1, Bmi1, Sox2 and Oct4 in the specified cell lines, following the procedure outlined by our group previously [17]. The primer sequences utilized in this analysis can be found in Tables S3 and S4. Further details can be found in the Supplementary Materials and Methods.

### Western blot

Western blot analysis was conducted to evaluate the expression of DNMT1, FoxM1, and CD44 proteins in the designated HCC cells, as previously outlined by our group [15]. Protein extraction was carried out using RIPA lysis buffer. Identical quantities of protein were separated by SDS-PAGE and subsequently transferred to the PVDF membrane. After blocking with 5% non-fat milk powder diluted in TBST, they were incubated with the specified antibodies: DNMT1 (1:1000 dilution), CD44 (1:1000 dilution), FoxM1 (1:1000 dilution), and β-actin GAPDH (1:2000 dilution). Following primary antibody incubation, the membranes were exposed to HRP-conjugated anti-rabbit IgG (1:2000 dilution) or HRP-conjugated anti-mouse IgG (1:2000 dilution) for 2 h at room temperature. Protein signals were then detected employing the enhanced chemiluminescence reagent (Roche).

### 3′UTR dual luciferase reporter assay

To investigate whether FoxM1 represents a target gene of miR-34a-5p in HCC cells, the 3'UTR dual luciferase reporter assay was conducted, as previously described by our group [18]. In brief, a dual luciferase reporter plasmid containing either the wild-type FoxM1 3'UTR or a mutated variant with modifications at the predicted miR-34a-5p binding sites was constructed by GeneCopoeia. HCC cells underwent co-transfection with the plasmid alongside either miR-34a-5p mimic or a control mimic (RiboBio) using Lipofectamine 2000 (Invitrogen). Following a 48 h incubation period, the cells were collected, and luciferase activity was assessed. Firefly and renilla luciferase activities were quantified using the Dual-Luciferase Reporter Assay System (Promega), and the final readings were obtained using a luminometer (Promega).

### Analysis of miR-34a-5p promoter methylation by pyrosequencing

Pyrosequencing was performed to determine DNA methylation levels in the promoter region of miR-34a-5p in the indicated cells. The three CpGs rich regions upstream from miR-34a-5p’s stem-loop sequence is shown in Fig. S1. The primer sequences utilized for the analysis were developed with the aid of Qiagen’s Pyromark Assay Design 2.0, as detailed in Table S5. The EZ DNA methylation kit (Zymo Research) was utilized for the detection of bisulfite modification. The target was amplified using specific primers by PCR. The reaction cycle was initiated with denaturation at 95 ℃ for 3 min, succeeded by 40 amplification cycles consisting of 30 s at 94 ℃, 30 s at 56 ℃, and 1 min at 72 ℃, then extension at 72 ℃ for 10 min using Qiagen PyroMark PCR kit. Following amplification, the target PCR product was sequenced on the PyroMark Q96 instrument (Qiagen). The pyrosequencing data, focusing on the incorporation of T and C nucleotides at the CpG sites, were analyzed using the CpG methylation analysis tool integrated within Qiagen’s PyroMark Q24 software. This software-generated output quantifies CpG methylation levels as a percentage, ranging from 0% (unmethylated) to 100% (fully methylated).

**Fig. 1:**
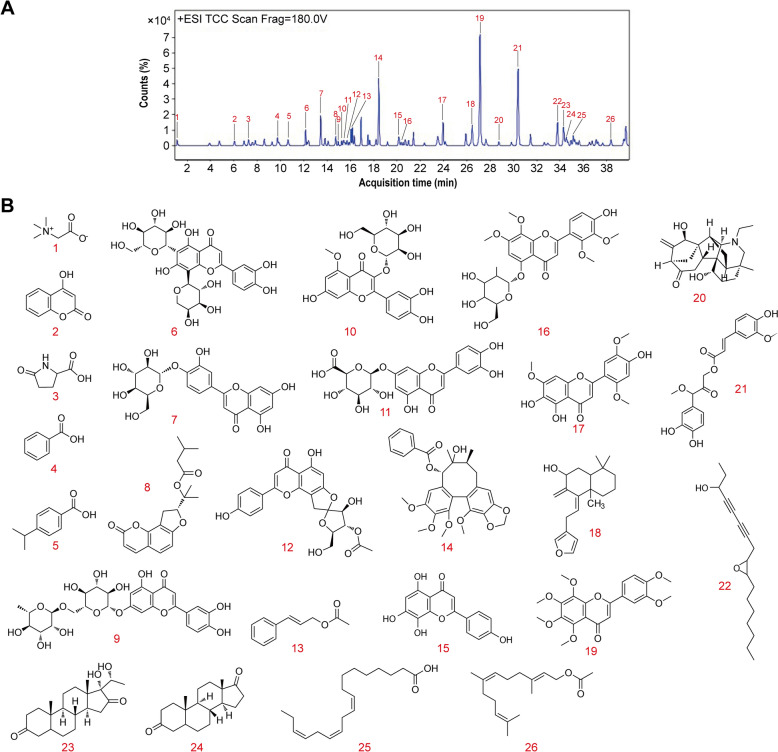
Identification of main chemical components in FVTF by UPLC-MS/MS. **A** A representative UPLC chromatograms of the 26 compounds in the FVTF. **B** The structure of 26 main chemical components in the FVTF

### Animal experiments

**Fig. 2:**
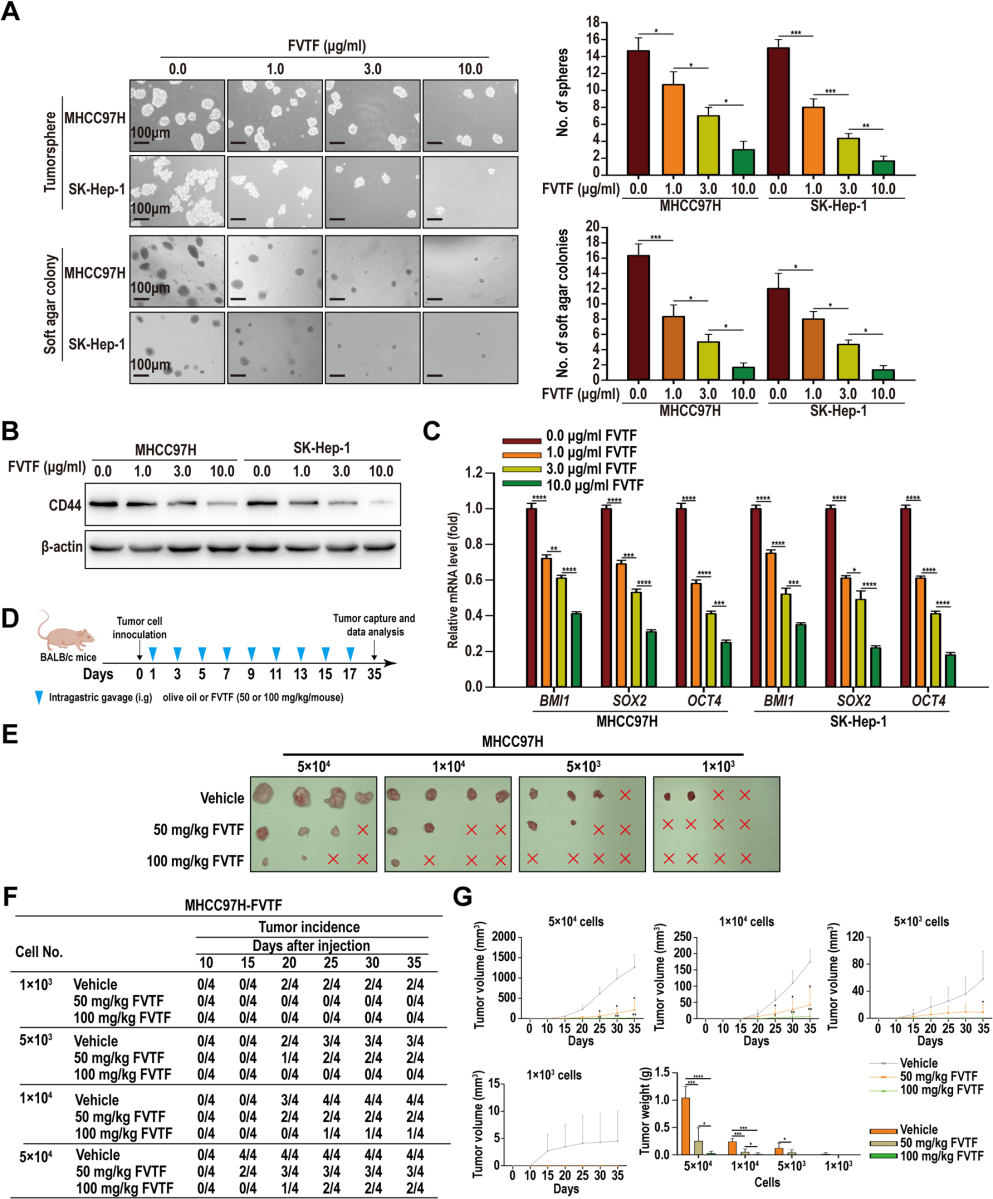
FVTF inhibits HCC cell stem properties in vitro and in vivo. MHCC97H and SK-Hep-1 HCC cells were treated with indicated concentrations of FVTF (0.0, 1.0, 3.0, 10.0 μg/ml), and subjected to in vitro cancer stem properties analyses. **A** Representative results (*left*) and statistical analysis (*right*) of tumorspheres and soft agar colonies formation in the HCC cells treated with FVTF. **B** Western blot analysis showing the expression levels of CD44 in the HCC cells treated with FVTF. **C** qRT-PCR showing the expression levels of *BMI1*, *SOX2* and *OCT4* in the HCC cells treated with FVTF. **D**–**G** FVTF decreases the tumor-initiating capacity of HCC cells in vivo. **D** Schematic diagram illustrating the treatment plan of FVTF in the nude mice with xenografts generated from MHCC97H cells. **E** The photographs of xenografts 35 days after initial FVTF treatment. **F** Summary data of tumor-initiation capacity of the HCC cells after initial FVTF treatment. **G** Tumor volume was periodically monitored and tumor growth curves of 1 × 10^3^, 5 × 10^3^, 1 × 10^4^ and 5 × 10^4^ HCC cells were plotted, and average tumor weight after initial FVTF treatment is also shown. Scale bars = 100 μm. ^*^P < 0.05; ^**^P < 0.01; ^**^P < 0.001; ^****^P < 0.0001; ns, no significance

To assess the effect of FVTF on the in vivo tumor-initiating capacity, tumorspheres of HCC MHCC97H cells were dissociated into single-cell suspension, serial dilutions of tumorsphere cells (5 × 10^4^, 1 × 10^4^, 5 × 10^3^, or 1 × 10^3^) were administered via subcutaneous injection into nude mice (n = 4 mice each group). After the inoculation, the mice were assigned at random to either the experimental or the control group (n = 4 mice each group). Mice in the experimental group received FVTF treatment (50 or 100 mg/kg once daily, 3 times one week for continuous 3 weeks) by intragastric gavage, and mice in the control group received the equivalent olive by intragastric gavage. The detailed schematic representation of the treatment plans is illustrated in Fig. 2D.

The mice were observed daily for the presence of palpable tumors. The tumor volume was calculated using caliper and the modified ellipsoid formula: (length × width^2^/2). After a period of five weeks, they were humanely euthanized by cervical dislocation, after that the subcutaneous xenografts were harvested. The tumor tissues underwent fixation in 4% paraformaldehyde and were then embedded in paraffin for the purposes of immunohistochemistry and in situ hybridization studies.

### Immunohistochemisry and in situ hybridization

Immunohistochemistry of DNMT1 and FoxM1, and in situ hybridization (ISH) of miR-34a-5p were conducted on the TMA. For additional details, see the Supplementary Materials and Methods.

### Statistical analysis

IBM SPSS 22 was employed for statistical analysis, while GraphPad Prism 8.0 was used to generate data visualizations. The data are presented as mean ± standard deviation (SD). Group comparisons were performed using Student's *t* test and/or one-way ANOVA, followed by Dunnett's tests. The Pearson correlation test was used to assess correlations. Kaplan–Meier curves were constructed for survival analysis, and the log-rank test was employed to evaluate statistical significance. Statistical significance was defined as a *P*-value lower than 0.05 (*P* < 0.05).

## Results

### Identification of main chemical components in FVTF by UPLC-MS/MS

FVTF's composition was analyzed by UPLC-MS/MS. Figure 1A showed a positive ionization chromatogram of a base peak chromatogram (BPC), various component peaks of which were analysed using mass spectrometry. A total of 26 compounds, comprising 10 flavones (Carlinoside, Luteolin-4′-beta-D-glucoside, o-Isovalerylcolum Bianetin, Luteolin-7-rutinoside, Luteolin-7-beta-D-glucuronide, Pinnatifin I, Isoscutellarein, Andrographin F, Isoarcapillin and Nobiletin), from FVTF were identified (Fig. 1B and Fig. S2, and Table S6). Among the identified compounds, compound 19 (Nobiletin) was found to be the most abundant in FVTF (Fig. 1A).

### FVTF inhibits HCC cell stem properties in vitro and in vivo

To investigate the effect of FVTF on the stem properties of HCC cells in vitro, MHCC97H and SK-Hep-1 HCC cells were subjected to various concentrations of FVTF (0.0, 1.0, 3.0 and 10.0 μg/ml), and subjected to analysis of cancer cell stem properties. The findings indicated that FVTF diminished tumorsphere and soft agar colony formation capacities, as well as the protein expression levels of CD44 and the mRNA of BMI1, OCT4, and SOX2 in a dose-dependent manner (Fig. 2A–C). To evaluate the impact of FVTF on tumor-initiating capacity of HCC cells, serial dilutions of MHCC97H cells (1 × 10^3^, 5 × 10^3^, 1 × 10^4^ or 5 × 10^4^) were subcutaneously administered to nude mice, while tumor-bearing mice received FVTF treatment (50 or 100 mg/kg once daily, and 3 times one week for continuous 3 weeks) by intragastric gavage (Fig. 2D). After initial treatment, the progression and expansion of the tumor were observed over a period of five weeks. The results indicated that FVTF inhibited the formation frequency and growth rate of tumors in a dose-dependent manner (Fig. 2E–G), revealing that FVTF significantly decreased the tumor-initiating capacity of HCC cells in vivo. Collectively, our results demonstrate that FVTF inhibits HCC cell stem properties in vitro and in vivo.

### FVTF targets inhibiting DNMT1/miR-34a-5p/FoxM1 axis in HCC cells

**Fig. 3:**
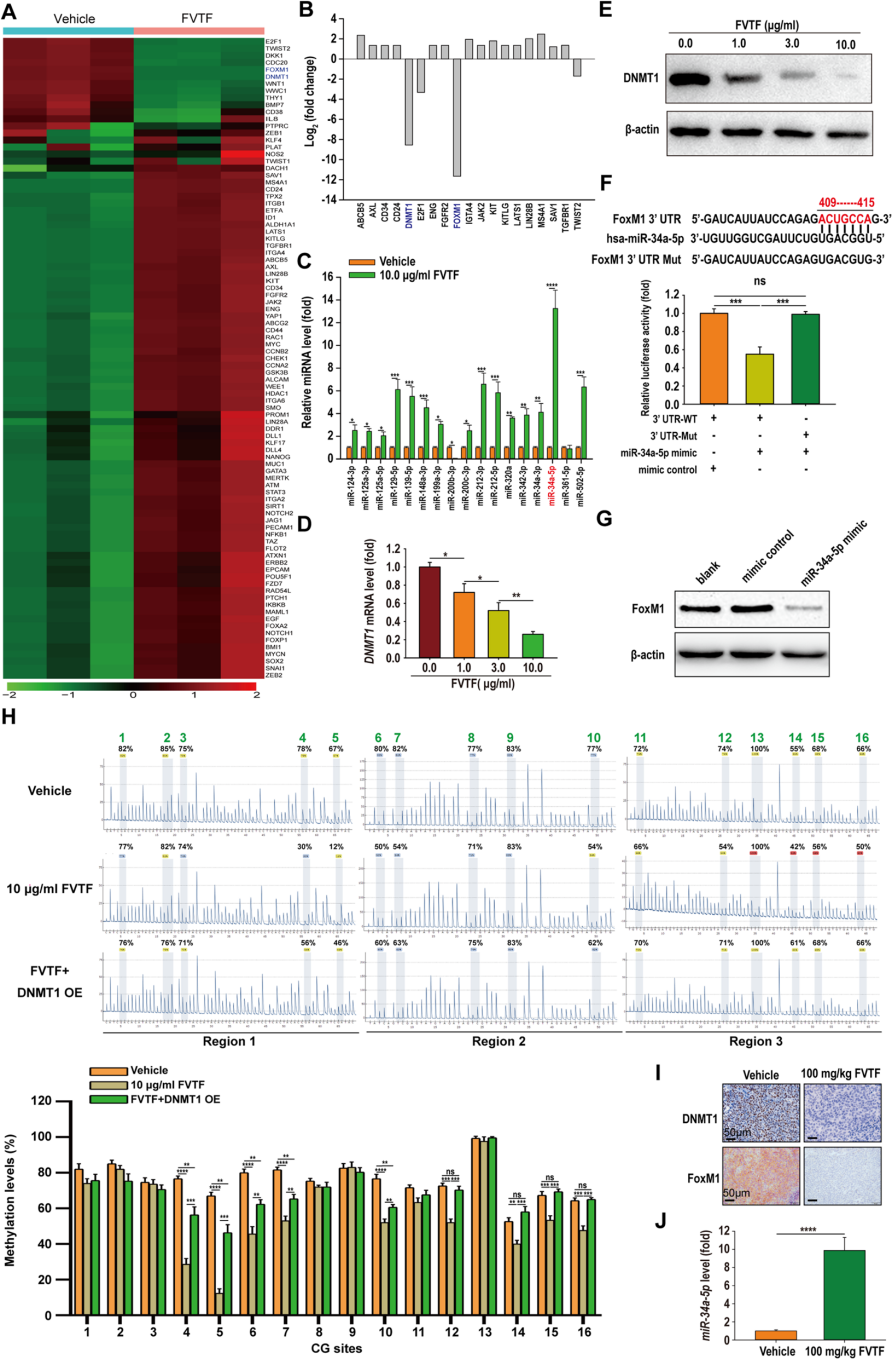
FVTF targets inhibiting DNMT1/miR-34a-5p/FoxM1 axis in HCC. **A** Heatmap of differential expression cancer stem cell-related genes in the FVTF-treated MHCC97H cells and control cells identified by PCR array. Each row represents a mRNA, and each column represents a sample. **B** Column chart showing 15 up-regulated mRNAs and 4 down-regulated mRNAs in the FVTF-treated MHCC97H cells identified by PCR array. **C** qRT-PCR showing the relative expression levels of 17 miRNAs in the FVTF-treated MHCC97H cells and control cells. **D**, **E** qRT-PCR (**D**) and Western blot analysis (**E**) showing the mRNA and protein expression levels of DNMT1 in the HCC cells treated with indicated concentrations of FVTF. **F** 3′UTR dual luciferase reporter assay. The predicted miR-34a-5p-binding sites in the wild-type (WT) FoxM1 3’ UTR and mutant (Mut) FoxM1 3’ UTR are shown (*top*); WT or Mut FOXM1 3’ UTR dual luciferase reporter vector was cotransfected into HCC cells with control or miR-34a-5p mimic, and firefly luciferase activity normalized to renilla luciferase activity is shown (*bottom*). **G** Western blot showing FoxM1 expression level in the HCC cells transfected with control or miR-34a-5p mimic. **H** Bisulfite pyrosequencing assay showing the methylation levels of miR-34a-5p promoter CG sites in the FVTF-treated HCC and DNMT1 OE cells. The representative results (*top*) and statistical analysis (*bottom*) of miR-34a-5p promoter methylation levels are shown. **I** Immunohistochemistry showing the expression levels of DNMT1 and FoxM1 in the xenografts with or without FVTF treatment. **J** qRT-PCR showing the expression levels of miR-34a-5p in the xenografts with or without FVTF treatment. Scale bars = 50 μm. ^*^P < 0.05; ^**^P < 0.01; ^**^P < 0.001; ^****^P < 0.0001; ns, no significance.

To examine the molecular mechanisms by which FVTF inhibits the stemness of HCC cells, PCR-array was used to screen differential expression cancer stem-related genes between the FVTF-treated and untreated MHCC97H cells. As a result, 15 upregulated and 4 downregulated mRNAs were identified in the FVTF-treated MHCC97H cells (Fig. 3A and B, and Table S7). Among the four downregulated mRNAs, FoxM1 and DNMT1 were downregulated 11.6541 and 8.56 folds respectively (Fig. 3B and Table S7), which were the most obviously downregulated two mRNAs. We also analyzed the expression changes of 17 cancer stem-related miRNAs between the FVTF-treated and untreated MHCC97H cells by qRT-PCR, and identified miR-34a-5p as the most obviously upregulated miRNA (Fig. 3C and Fig. S3). Together, the results indicate that DNMT1, miR-34a-5p and FoxM1 could be the targets of FVTF in the HCC cells.

Next, the impact of FVTF on DNMT1 expression in HCC cells was validated, revealing that FVTF downregulated both mRNA and protein levels of DNMT1 in a dose-dependent manner (Fig. 3D and E). This indicates that DNMT1 could be a direct target of FVTF in HCC cells. MiRNAs are intrinsic short non-coding RNAs that are essential in gene regulation via their interaction with the 3' untranslated region of target mRNAs. Since FVTF upregulated miR-34a-5p and downregulated both FoxM1 and DNMT1, we predict whether FoxM1 and DNMT1 are the targets of miR-34a-5p using miRWalk database (http://mirwalk.umm.uni-heidelberg.de/). The result showed FoxM1 was a putative target gene of miR-34a-5p (Fig. 3F), but DNMT1 was not its target gene (data not shown). To validate FoxM1 as a direct target of miR-34a-5p, we co-transfected HCC cells with a dual luciferase reporter plasmid containing the wild-type FoxM1 3'UTR and either a control mimic or miR-34a-5p mimic. The findings indicated a notable decrease in luciferase activity in cells transfected with the miR-34a-5p mimic relative to those transfected with the control mimic. Conversely, the miR-34a-5p mimic did not significantly affect the luciferase activity of a dual luciferase reporter plasmid containing a mutant FoxM1 3' UTR, where the miR-34a-5p binding site was modified (Fig. 3F). Furthermore, FoxM1 levels were substantially decreased in the miR-34a-5p mimic-transfected HCC cells (Fig. 3G). Collectively, our findings indicate that FoxM1 represents a direct target of miR-34a-5p, and that FVTF reduces FoxM1 expression via upregulating miR-34a-5p in HCC cells.

MiRNA expression is also affected by epigenetic regulation such as promoter methylation. To explore whether FVTF affects methylation of miR-34a-5p, we detected the DNA methylation frequencies of miR-34a-5p promoter in FVTF-treated MHCC97H cells by pyrosequencing, and found that methylation frequencies of miR-34a-5p promoter CG sites were substantially reduced in FVTF-treated HCC cells compared to vehicle-treated control cells (Fig. 3H), indicating that FVTF reduces miR-34a-5p expression by promoter demethylation. Since DNMT1 serves as a crucial element within the epigenetic framework, influencing gene repression by increasing promoter methylation levels, we explored whether DNMT1 mediates the FVTF-downregulating miR-34a-5p promoter methylation, and found that overexpression of DNMT1 restored the methylation levels of most of miR-34a-5p promoter CG sites in the FVTF-treated HCC cells (Fig. 3H), indicating that FVTF demethylates miR-34a-5p promoter by downregulating DNMT1 expression. Moreover, FVTF also decreased the expression levels of DNMT1 and FoxM1, and increased miR-34a-5p expression level in the subcutaneous xenografts of mice (Fig. 3I and J). Taken together, our results suggest that FVTF targets inhibiting DNMT1/miR-34a-5p/FoxM1 axis in HCC cells.

### *FVTF inhibits HCC cell stemness *via* targeting DNMT1/miR-34a-5p/FOXM1 axis*

**Fig. 4:**
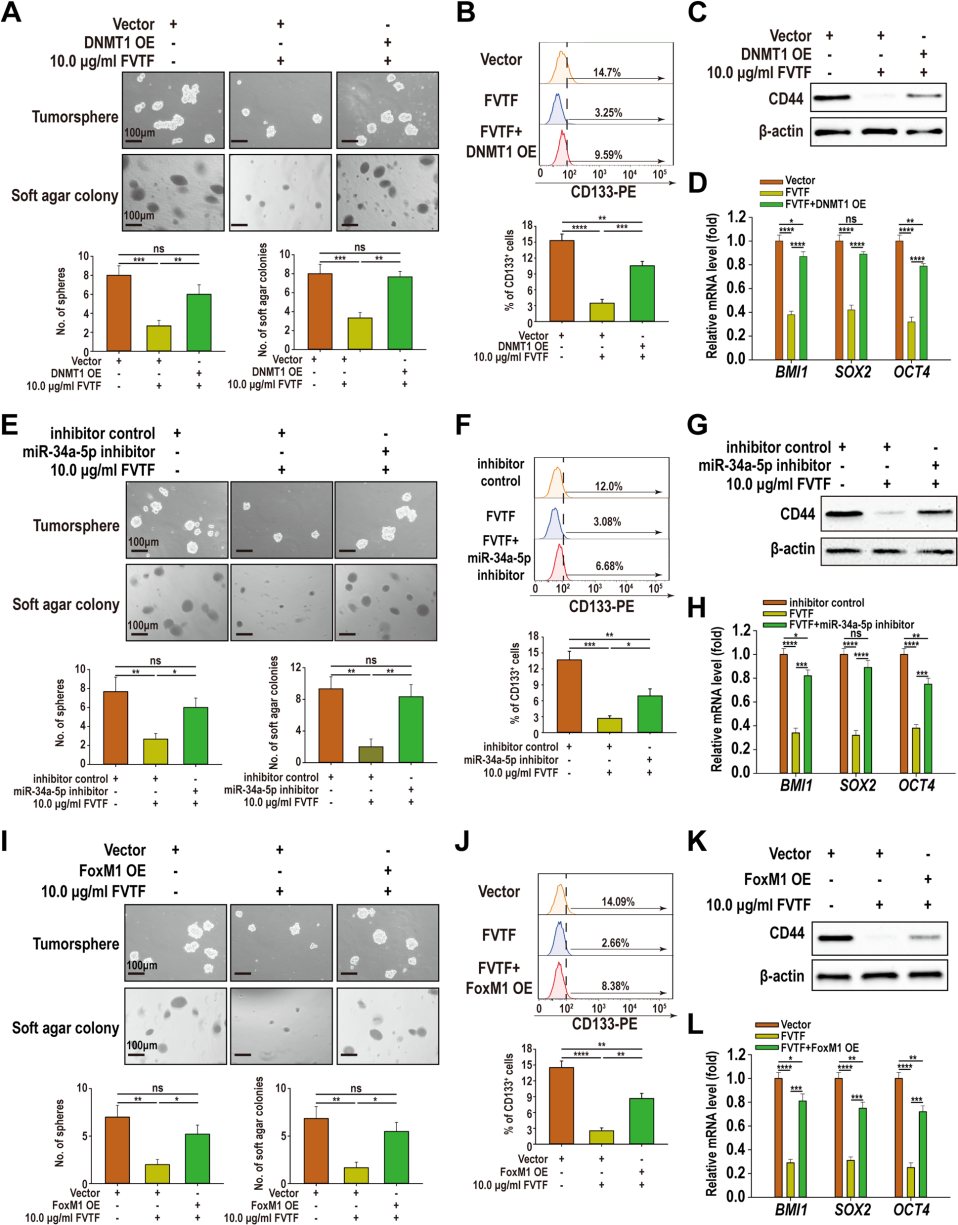
FVTF inhibits HCC cell stemness via targeting DNMT1/miR-34a-5p/FoxM1 axis. **A**–**D** DNMT1 overexpression (OE) antagonizes the effect of FVTF on formation ability of tumorsphere and soft agar colony (**A**), the percentage of CD133 positive cells (**A**), and the expression levels of CD44 protein (**C**) and *BMI1*, *OCT4* and *SOX2* mRNA (**D**) in the MHCC97H cells. **E**–**H** miR-34a-5p inhibitor antagonizes the effect of FVTF on formation ability of tumorsphere and soft agar colony (**E**), the percentage of CD133 positive cells (**F**), and the expression levels of CD44 protein (**G**) and *BMI1*, *OCT4* and *SOX2* mRNA (**H**) in the MHCC97H cells. **I**–**L** FoxM1 OE antagonizes the effect of FVTF on formation ability of tumorsphere and soft agar colony (**I**), the percentage of CD133 positive cells (**J**), and the expression levels of CD44 protein (**K**) and *BMI1*, *OCT4* and *SOX2* mRNA (**L**) in the MHCC97H cells. Scale bars = 100 μm. ^*^P < 0.05; ^**^P < 0.01; ^**^P < 0.001; ^****^P < 0.0001; ns, no significance

To explore whether DNMT1 is involved in FVTF-inhibiting HCC cell stemness, we established MHCC97H cell line with DNMT1 overexpression (OE) (Fig. S4), and observed that DNMT1 OE was able to antagonize the effect of FVTF on formation ability of tumorsphere and soft agar colony, the percentage of CD133 positive cells, and the levels of CD44 protein and *BMI1*, *OCT4* and *SOX2* mRNA in the MHCC97H cells (Fig. 4A–D), indicating that FVTF inhibits HCC cell stemness by decreasing DNMT1 expression. Moreover, as DNMT1 was not the target of miR-34a-5p, and FoxM1 overexpression had also not affected on DNMT1 expression (Fig. S5A and B), indicating that FVTF inhibits HCC cell stemness via directly targeting DNMT1.

To explore whether miR-34a-5p is involved in FVTF-inhibiting HCC cell stemness, we transfected miR-34a-5p inhibitor into MHCC97H cells, and observed that miR-34a-5p inhibitor could antagonize the effect of FVTF on the formation ability of tumorsphere and soft agar colony, the percentage of CD133 positive cells, and the levels of CD44 protein and *BMI1*, *OCT4* and *SOX2* mRNA in the MHCC97H cells (Fig. 4E–H), suggesting that FVTF inhibits HCC cell stemness by upregulating miR-34a-5p. Moreover, we observed that DNMT1 overexpression was able to antagonize the influence of FVTF on miR-34a-5p expression (Fig. S5C), supporting that FVTF inhibits HCC cell stemness via targeting DNMT1/miR-34a-5p axis.

To explore whether FoxM1 is involved in FVTF-inhibiting HCC cell stemness, we established MHCC97H cell line with FoxM1 overexpression (Fig. S4), and observed that FoxM1 overexpression was able to antagonize the effect of FVTF on formation ability of tumorsphere and soft agar colony, the percentage of CD133 positive cells, and the levels of CD44 protein and *BMI1*, *OCT4* and *SOX2* mRNA in the MHCC97H cells (Fig. 4I-L), indicating that FVTF inhibits HCC cell stemness by reducing FoxM1 expression. Moreover, we observed that DNMT1 overexpression or miR-34a-5p inhibitor was able to antagonize the effect of FVTF on FoxM1 expression (Fig. S5D and E), supporting that FVTF inhibits HCC cell stemness via targeting DNMT1/miR-34a-5p/FoxM1 axis.

### Expression correlation and clinicopathological significance of DNMT1, miR-34a-5p and FoxM1 in HCC tissues

**Fig. 5:**
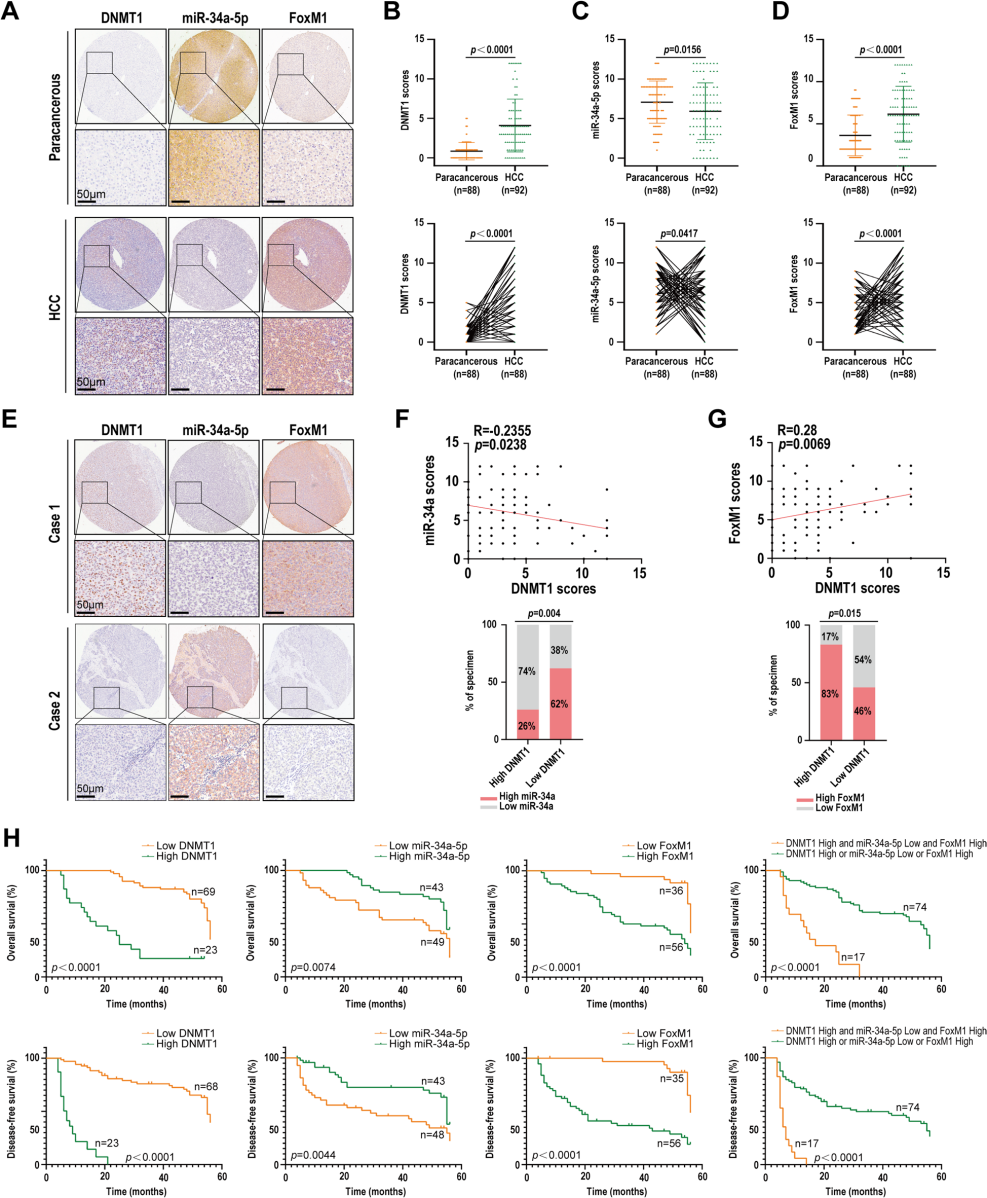
Expression correlation and clinicopathological significance of DNMT1, miR-34a-5p and FoxM1 in HCC tissues. **A**–**D** Immunohistochemistry and in situ hybridization showing the expression level of DNMT1 and FoxM1, and miR-34a-5p in the HCC and paired paracancerous tissues respectively. **A** Representative images of DNMT1, miR-34a-5p and FoxM1 expressions, and (**B**–**D**) Statistical analysis results of DNMT1, miR-34a-5p and FoxM1 expressions. **E**–**G** Correlation between alterations for DNMT1, miR-34a-5p, and FoxM1 in the HCC tissues. **E** Representative IHC images of low and high expression of DNMT1, FoxM1, and miR-34a-5p the HCC tissues. **F**, **G** Statistical analysis results of correlation between DNMT1 and miR-34a-5p expressions, and miR-34a-5p and FoxM1 expressions (Pearson correlation test). **H** Survival analysis of HCC patients. Kaplan–Meier survival analysis of overall survival (*top*) and disease-free survival (*bottom*) for 92 HCC patients based on the expression levels of DNMT1, miR-34a-5p, FoxM1, or a combination of three molecules. The log-rank test was used to calculate the *P* value. Scale bars = 50 μm

To investigate the clinical implications of DNMT1, miR-34a-5p, and FoxM1 expression in the patients with HCC, we detect DNMT1 and FoxM1 expression by immunohistochemical staining, and miR-34a-5p expression using in situ hybridization in the HCC tissue microarray (TMA) containing 92 HCC tissues and 88 paired paracancerous tissues. The results indicated that HCC tissues had significantly higher expression levels of DNMT1 and FoxM1 compared to paracancerous tissues, whereas the expression of miR-34a-5p was dramatically diminished relative to paracancerous tissues (Fig. 5A–D). The expression level of DNMT1 showed an inverse correlation with that of miR-34a-5p, while it exhibited a positive correlation with the level of FoxM1 (Fig. 5E–G), and high DNMT1 levels, low miR-34a-5p levels, and high FoxM1 levels were correlated with cancer recurrence in the HCC patients (Table S2). Survival analysis showed that patients with DNMT1^High^, miR-34a-5p^Low^ and FoxM1^High^ expression experienced reduced overall survival (OS) and disease-free survival (DFS) relative to patients with DNMT1^Low^, miRNA-34-5p^High^ or FoxM1^Low^ expression (Fig. 5H). A univariate and multivariate Cox regression analysis indicated that the combination of DNMT1, miR-34a-5p, and FoxM1 serves as a distinct marker for both DFS and OS (Table S8). Collectively, our findings suggest that DNMT1/miR-34a-5p/FoxM1 axis may be pivotal in HCC recurrence, and a combination of DNMT1, miR-34a-5p-5p and FoxM1 may function as a potential marker for prognostic prediction in individuals diagnosed with HCC.

## Discussion

Many studies have shown that certain phytomedicines possess active molecules with anti-cancer effects. Consequently, the extraction and isolation of anti-cancer compounds from naturally existing plants has emerged as a pivotal method in the identification of novel anti-tumor pharmaceuticals. In this study, we extracted and prepared FVTF from Fructus Viticis, identified a total of 26 compounds, including 10 flavones. Prior investigations have confirmed that cancer stem-like cells (CSLCs) exist within HCC cells [4, 5]. CSLCs significantly influence the occurrence and progression of HCC, being intricately linked to aspects such as HCC invasion, metastasis, drug resistance, and recurrence following treatment [19]. Therefore, the ability to form tumorspheres or colonies in soft agar, the expression levels of several known HCC stem markers (OCT4, SOX2, BMI1) [20, 21], the percentage of HCC stem marker CD133^+^ cells [22], and a xenograft model were employed to assess the effect of FVTF on HCC stem properties. Our data reveal that FVTF inhibits in vitro and in vivo HCC cell stem properties, and FVTF inhibits HCC cell stemness via targeting DNMT1/miR-34a-5p/FoxM1 axis, indicating that FVTF acts as a therapeutic preparation against HCC. We also found that DNMT1/miR-34a-5p/FoxM1 axis may be pivotal in HCC recurrence, and a combination of DNMT1, miR-34a-5p and FoxM1 could function as a prognostic indicator in HCC patients.

Aberrant DNA methylation, catalyzed by DNMT1, is associated with various cancers by silencing of tumor suppressor genes, and is identified as a significant contributor to the advancement of cancer [23]. DNMT1 is an important epigenetic target for drug development since the DNA methylation is reversible [24]. Although DNMT1 inhibitor, Decitabine, has received FDA approval for the treatment of leukemia, discovering more DNMT1 inhibitors for cancer therapy is needed. This study demonstrates that FVTF substantially decreased in vitro and in vivo stemness of HCC cells through the downregulation of DNMT1, indicating that FVTF may be a promising anti-cancer agent via targeting DNMT1.

A total of ten flavonoid compounds, including carlinoside, luteolin-4'-beta-D-glucoside, o-isovalerylcolum bianetin, luteolin-7-rutinoside, luteolin-7-beta-D-glucuronide, pinnatifin I, isoscutellarein, andrographin F, isoarcapillin and nobiletin, were identified in FVTF. Flavonoid luteolin has been found to promote cell cycle arrest and death in HeLa cells through reducing DNMT1 expression [25], and luteolin also inhibits intracellular methylation activity of breast cancer cells [26], flavonoid nobiletin enhances the efficacy of chemotherapy through targeting colorectal cancer stem cells [27], and flavonoid nobiletin attenuates hepatocyte growth factor (HGF)-induced liver cancer cells metastasis [28]. Since FVTF contains ten flavonoid compounds, we reasonably think that FVTF’s anti-cancer activity is better than flavonoid monomer.

MiR-34a-5p is an important tumor suppressor miRNA, and has a potential for treating cancers [29]. Luteolin was discovered to upregulate miR-34a expression in human gastric [30] and non-small cell lung cancer cells [31]. This study demonstrates that FVTF induces demethylation of miR-34a-5p, while enhances its expression through downregulating DNMT1 expression. These data indicate that FVTF, as an epigenetic regulating agent for miR-34a-5p, is an appealing option for cancer therapy.

FoxM1 is an important transcription factor in cancer stem cells, playing a significant role in various cancers [32]. It has been reported that FoxM1 can increase CD44 expression via binding with CD44 promoter in human liver cancer cells [33]. Moreover, FoxM1 can upregulate the expression of cancer stemness genes, such as BMI1, NANOG, and c-MYC, in HCC cells [34]. In this research, we demonstrated that FVTF reduces FoxM1 expression via upregulating miR-34a-5p in HCC cells, indicating that FVTF-inhibiting HCC stemness is closely associated with its downregulating FoxM1 expression.

We found that FVTF inhibits HCC cell stemness via targeting DNMT1/miR-34a-5p/FoxM1 axis. Consistent with this observation, the expression levels of DNMT1 and FoxM1 were substantially elevated in HCC tissues compared to adjacent non-cancerous tissues, whereas the expression of miR-34a-5p was notably reduced in HCC tissues relative to paracancerous tissues. Furthermore, DNMT1 exhibited a negative correlation with miR-34a-5p levels and a positive correlation with FoxM1 expression; and alterations in DNMT1, miR-34a-5p, and FoxM1 expression were associated with HCC recurrence, suggesting that DNMT1/miR-34a-5p/FoxM1 axis is pivotal in HCC and represents an important therapeutic target in HCC. Moreover, our data reveal that a combination of DNMT1, miR-34a-5p and FoxM1 could function as a potential prognostic prediction marker in patients diagnosed with HCC.

## Conclusion

In summary, our current investigation demonstrates that FVTF inhibits HCC cell stem properties by targeting DNMT1/miR-34a-5p/FoxM1 axis, and dysregulation of DNMT1, miR-34a-5p and FoxM1 expression is correlated with HCC recurrence and prognosis. Our observations suggest that FVTF may function as a prospective therapeutic agent for human HCC, while a combination of DNMT1 and miR-34a-5p and FoxM1 could serve as a prognostic marker in HCC patients.

## Data Availability

The data and materials supporting the current study are available from the corresponding author upon reasonable request.

## Abbreviations

BPC: Base peak chromatogram
BSA: Bovine serum albumin
CSLCs: Cancer stem like cells
DFS: Disease-free survival
DMEM: Dulbecco’s modified eagle’s medium
DNMT1: DNA methyltransferase 1
FoxM1: Forkhead box protein M1
FVTF: Fructus viticis total flavonoids
GAPDH: Glyceraldehyde-3-phosphate dehydrogenase
HCC: Hepatocellular carcinoma
ISH: In situ hybridization
LC: Liquid chromatography
miRNA: MicroRNA
MS: Mass spectrometer
OCT4: Octamer-binding transcription factor 4
OS: Overall survival
qRT-PCR: Quantitative reverse transcription-polymerase chain reaction
Sox2: SRY-Box transcription factor 2
STR: Short tandem repeat
TMA: Tissue microarray
UPLC: Ultra-high performance liquid chromatography
UTR: Untranslated regions
